# Upper and Lower Limb Motor Function Correlates with Ipsilesional Corticospinal Tract and Red Nucleus Structural Integrity in Chronic Stroke: A Cross-Sectional, ROI-Based MRI Study

**DOI:** 10.1155/2021/3010555

**Published:** 2021-11-11

**Authors:** Denise M. Peters, Julius Fridriksson, Jessica D. Richardson, Jill C. Stewart, Chris Rorden, Leonardo Bonilha, Addie Middleton, Stacy L. Fritz

**Affiliations:** ^1^Department of Rehabilitation and Movement Science, University of Vermont, 106 Carrigan Dr., Burlington, VT, USA; ^2^Department of Communication Sciences and Disorders, University of South Carolina, 915 Greene St., Columbia, SC, USA; ^3^Department of Speech and Hearing Sciences, University of New Mexico, 1700 Lomas Blvd., Albuquerque, NM, USA; ^4^Department of Exercise Science, Physical Therapy Program, University of South Carolina, 921 Assembly St., Columbia, SC, USA; ^5^Department of Psychology, University of South Carolina, 1512 Pendleton St., Columbia, SC, USA; ^6^Department of Neurology, Medical University of South Carolina, 96 Jonathan Lucas St., Charleston, SC, USA; ^7^New England Geriatric Research Education and Clinical Center, VA Boston Healthcare System, 150 South Huntington Ave., Jamaica Plain, MA, USA

## Abstract

**Background:**

Structural integrity of the ipsilesional corticospinal tract (CST) is important for upper limb motor recovery after stroke. However, additional neuromechanisms associated with motor function poststroke are less well understood, especially regarding the lower limb.

**Objective:**

To investigate the neural basis of upper/lower limb motor deficits poststroke by correlating measures of motor function with diffusion tensor imaging-derived indices of white matter integrity (fractional anisotropy (FA), mean diffusivity (MD)) in primary and secondary motor tracts/structures.

**Methods:**

Forty-three individuals with chronic stroke (time poststroke, 64.4 ± 58.8 months) underwent a comprehensive motor assessment and MRI scanning. Correlation and multiple regression analyses were performed to examine relationships between FA/MD in a priori motor tracts/structures and motor function.

**Results:**

FA in the ipsilesional CST and red nucleus (RN) was positively correlated with motor function of both the affected upper and lower limb (*r* = 0.36‐0.55, *p* ≤ 0.01), while only ipsilesional RN FA was associated with gait speed (*r* = 0.50). Ipsilesional CST FA explained 37.3% of the variance in grip strength (*p* < 0.001) and 31.5% of the variance in Arm Motricity Index (*p* = 0.004). Measures of MD were not predictors of motor performance.

**Conclusions:**

Microstructural integrity of the ipsilesional CST is associated with both upper and lower limb motor function poststroke, but appears less important for gait speed. Integrity of the ipsilesional RN was also associated with motor performance, suggesting increased contributions from secondary motor areas may play a role in supporting chronic motor function and could become a target for interventions.

## 1. Introduction

Motor weakness is one of the most disabling consequences of stroke, often leading to difficulties in activities of daily living, gait, and decreased activity levels [1]. Recovery of motor function varies considerably, as patients with similar lesions on structural scans can exhibit different motor impairments and/or responses to treatments. Motor recovery depends on adaptive processes in both the affected and unaffected hemisphere, although the exact neural mechanisms remain unclear [2, 3].

Diffusion tensor imaging (DTI) allows for the examination of the integrity and orientation of white matter in the brain by estimating the magnitude and directionality of water diffusion [4]. Two of the most common DTI-derived metrics are mean diffusivity (MD), which represents the overall magnitude of water diffusion, and fractional anisotropy (FA), which reflects the degree of diffusion directionality [5]. Loss of microstructural integrity of white matter tracts (e.g., local tissue damage within the primary lesion, anterograde, and/or retrograde axonal degeneration) is typically reflected by an increase in MD (representing increased water diffusion in the extracellular space) and/or a reduction in FA (representing decreased anisotropic diffusion) [5]. Axonal properties such as density, myelination, diameter, and orientation contribute to overall FA values [6].

Several studies using DTI techniques have demonstrated a correlation between upper limb motor dysfunction and decreased integrity of white matter tracts in both acute [7, 8] and chronic [9–11] stroke. Reduced structural damage to the ipsilesional corticospinal tract (CST) is associated with better motor outcome after stroke [9, 12], yet a significant amount of variance remains unexplained. While the CST is the main motor pathway for voluntary movements of the hand, secondary motor pathways and brain regions also play a role in upper limb motor function and recovery. Microstructural changes in transcallosal fibers (i.e., higher FA values) have also been associated with better motor function poststroke [9, 11]. Additionally, neuroplastic changes have been shown to occur in the red nucleus (RN) of the affected [13, 14] and unaffected hemisphere [14]. The RN (located in the rostral midbrain) is the origin of the rubrospinal tract (RST) [15]. The RST and CST are functionally related with their fibers terminating in close proximity in the spinal cord, suggesting the RN may have some potential to compensate for CST injury following stroke. Studies have suggested that anisotropy within deep nuclear structures (such as the RN) may be related to the axon bundles traveling within them [16], and that increased FA values in such nuclei could indicate remodeling and neuroplastic changes after stroke [13, 17].

Motor recovery of the affected upper limb has been highly studied whereas less is known about the neuromechanisms involved with lower limb function after stroke. While the CST is necessary for fine movements of the hands [18, 19], locomotion and motor function of the legs is less dependent on the CST [20, 21]. Greater structural damage to the CST has been associated with decreased knee extensor strength [22], decreased dorsiflexion and hip flexion movement [23], and increased walking impairment [23, 24] in chronic stroke. On the other hand, locomotor ability is present in some stroke survivors despite complete lateral CST injury [20, 21]. In older adults, decreased white matter integrity in the internal capsule and corpus callosum has been associated with gait impairment [25], yet these relationships need further exploration in individuals with stroke.

Overall, these finding suggest that while the integrity of descending neural pathways from the ipsilesional motor system is important for predicting chronic motor function after stroke, contributions from secondary motor areas and tracts may also play a role. Most studies to date have had small sample sizes (*n* < 20), used outcome measures that are not clinically feasible or do not capture different International Classification of Functioning, Disability, and Health (ICF) domains, or focused solely on the CST. Few studies have included measures of subcortical structures or examined chronic white matter integrity and lower limb motor function or gait [24]. We previously reported that cortical disconnection of the ipsilesional primary motor cortex is associated with both gait speed and upper limb motor deficits in chronic stroke [26]. Scalar diffusion parameters such as FA and MD, however, are more efficient to obtain (in terms of acquisition and postprocessing) than connectivity metrics, making their clinical utility more appealing. Therefore, the aim of the present study was to investigate the neural basis of both upper and lower limb motor deficits in a larger sample of individuals with stroke by correlating measures of motor function with DTI-derived indices of white matter integrity (FA and MD) in primary and secondary motor tracts/regions. We hypothesized that (1) poor upper and lower limb motor function would be correlated with reduced white matter integrity (i.e., lower FA and/or higher MD values) in the ipsilesional CST and corpus callosum, and (2) microstructural changes (as indicated by higher FA values) in secondary motor control regions would be positively associated with upper and lower limb motor performance.

## 2. Methods

### 2.1. Participants

The same cohort of individuals with chronic stroke involved in our previous connectivity analysis [26] was included in the current analysis (see prior publication for participant demographic, behavioral, and lesion overlay data; https://pericles.pericles-prod.literatumonline.com/doi/epdf/10.1002/hbm.23829). Participants were recruited from January 2013 to June 2014. Eligibility criteria included single left hemispheric stroke ≥6 months (with no maximal time post-stroke cut-off), able to follow simple instructions, and able to independently walk 8 meters with or without an assistive device. Potential participants were excluded if they had contraindications to MRI, clinically reported history of dementia or other neurological condition (e.g., Parkinson's Disease), or extensive visual problems. All participants provided written informed consent approved by the Institutional Review Board at the University of South Carolina.

### 2.2. Motor Assessment

All participants underwent a comprehensive behavioral assessment of upper and lower limb motor function and gait at our university research lab. Testing was performed by a physical therapist and included the Box and Block Test (BBT) [27] to assess gross manual dexterity of the hand, grip strength [28], Motricity Index (MI) [29] to examine motor function/strength of the upper and lower limbs, and gait speed [30]. Participants used their typical assistive device for the gait speed assessment.

### 2.3. MRI Acquisition

All participants underwent scanning using a 3 T Siemens Trio system with a 12-element head coil at the McCausland Center for Brain Imaging (Columbia, SC) within two days of behavioral testing. High-resolution 3D T_1_-MRI scans (repetition time (TR) = 2250 ms, inversion time (TI) 925 ms, echo time (TE) = 4.15 ms, flip angle = 9°, field of view (FOV) = 256 mm, and voxel size = 1.0 × 1.0 × 1.0 mm) and 3D T_2_-MRI scans (TR = 3200 ms, TE = 212 ms, variable flip angle, FOV = 256 mm, and voxel size = 1.0 × 1.0 × 1.0 mm) were acquired for determination of lesion size and location. Diffusion imaging was performed with a single-shot gradient echo planar imaging (EPI) monopolar Stejskal-Tanner sequence using the following parameters: TR = 4987 ms, TE = 79.2 ms, flip angle = 90°, FOV = 207 mm, voxel size = 2.3 × 2.3 × 2.3 mm, slice thickness = 2.3 mm, 36 volumes with noncollinear diffusion directions at a *b* value of 1000 s/mm^2^ as well as 5 volumes with a *b* value of 0, and number of slices = 50. Slices were acquired with full Fourier and in-plane parallel acceleration (GRAPPA = 2). This diffusion sequence was acquired twice, with the second series reversing the phase-encoding direction.

### 2.4. Image Preprocessing

Stroke lesions were manually outlined by a neurologist (Bonilha, who was blinded to motor scores) on the T2 image, which was then coregistered to the T1 image. T1-weighted images were normalized into standard MNI space utilizing enantiomorphic unified segmentation-normalization routines as part of the software Statistical Parametric Mapping (SPM) 12 [31], which also applied a lesion-mask cost function [32]. Diffusion images were undistorted using FSL's TOPUP and Eddy tools [33, 34] with excess scalp removed using the FSL BET tool. FSL's dtifit tool was used to compute voxelwise maps of FA and MD. In order to improve registration between T1 and DTI spaces, the scalp-stripped (based on segmentation estimates) T1 image was nonlinearly normalized (using SPM12's “old normalization” function) to match the undistorted FA/MD images.

Region of interest (ROI) analyses were performed using the John Hopkins University (JHU) template overlaid on the voxelwise skeletons of FA and MD. ROIs included bilateral CST, body of the corpus callosum, red nuclei, substantia nigra, thalamus, and superior cerebellar peduncle. We focused our analyses on these ROIs as the CST is the major descending motor pathway and the body of the corpus callosum includes callosal motor fibers connecting cortex motor regions [35]. Furthermore, previous studies have highlighted associations between FA in the body of the corpus callosum and red nucleus and motor performance following stroke [10, 17, 36–38]. We also wanted to elucidate microstructural changes in specific subcortical regions involved in motor control (e.g., substantia nigra, thalamus, and superior cerebellar peduncle) and their relationship with motor function poststroke. The substantia nigra is part of the basal ganglia, a collection of nuclei with extensive brain connections important for postural control and voluntary movement, while the thalamus is a key relay station for sensory-motor neuronal loops involving the cerebellum and basal nuclei [15]. Mean FA and MD values were calculated in each ROI within the JHU atlas. These DTI parameters were chosen due to their commonality in assessing white matter integrity in prior stroke studies and for the different mechanisms they represent (i.e., FA reflects degree of diffusion directionality while MD represents the overall magnitude of water diffusion) [5]. Statistical analyses were performed on ROIs with a lesion load of <5% to minimize the influence of necrotic tissue on FA/MD values. FA values range from 0 to 1, with higher values indicating greater structural integrity/directionality.

### 2.5. Statistical Analyses

Differences in motor scores between affected/unaffected extremities were evaluated using two-tailed paired *t-*tests (or Wilcoxon signed-rank tests for nonparametric data). Spearman's correlation analyses were performed to examine the relationship between FA/MD in a priori motor tracts (bilateral CST (at the level of the pons) and body of the corpus callosum) and brain regions (bilateral red nuclei, substantia nigra, thalamus, and superior cerebellar peduncle) and upper/lower limb motor function. Correlations were interpreted as poor (<0.25), fair (0.25 to <0.5), moderate (0.5 to 0.75), and strong (>0.75) [39].

Regions of interest with a significant bivariate correlation (*p* ≤ 0.01) were entered into regression models to assess the amount of variance in upper and lower limb motor performance explained by the integrity of each ROI. In order to normalize data for regression analyses, participants who scored 0 on BBT were removed as the BBT has a low floor effect [27]. Similarly, participants who scored 99 on the MI were removed due to the high ceiling effect [29]. Separate multiple regressions were performed for each behavioral measure. Age and/or time since stroke were controlled for as covariates if significantly correlated with motor performance. Significance level was set at *p* < 0.05, corrected based on the number of behavioral measures assessed (corrected *p* ≤ 0.01).

An exploratory analysis was performed to investigate if DTI metric-motor behavior relationships differed based on level of motor severity. For upper extremity analyses, BBT scores were used to divide the sample into high (able to move ≥80% of blocks with paretic hand versus nonparetic) and low (able to move <80% of blocks) functioning groups. For lower extremity analyses, a gait speed of <0.8 meters/sec was used as the cut-off for high/low functioning groups which is the cut-off for community ambulator classification [40].

## 3. Results

### 3.1. Participants

Forty-three individuals with chronic stroke (mean age 59.7 ± 11.2 years; time post-stroke 64.4 ± 58.8 months; 38 persons right-hand dominant) participated in this study. Power analyses revealed a sample size of 37 would be sensitive (at 80% power, alpha of 0.05) to detect a correlation of 0.4 between upper limb function and ipsilesional CST FA using a bivariate correlation (one-tailed test). All participants exhibited a cortical/subcortical lesion in the left hemisphere, broadly distributed within the territory of the middle cerebral artery. Locations of maximal lesion overlap were the left extranuclear and subgyral areas.

### 3.2. Motor Performance

Motor performance of the affected extremities was significantly reduced compared to the unaffected extremity. The median BBT score for the affected upper limb was 34.5 [interquartile range (IQR), 7.25-46.5] and for the unaffected limb was 51.0 (IQR, 44.0-56.25) (*p* < 0.001). Average grip strength for the affected hand was 23.46 ± 15.72 kg and for the unaffected hand was 34.64 ± 10.51 kg (*p* < 0.001). The median Motricity Index score for the affected upper and lower limb was 88 (IQR, 56.5-100) and 79 (IQR, 59-100), respectively, and for the unaffected limbs was 100 (IQR, 100-100) (*p* < 0.001). Average gait speed was 0.94 ± 0.31 m/s. Participants, on average, exhibited a mild to moderate degree of motor impairment.

For the subgroup of participants for the regression analyses, average BBT score for the affected upper limb was 37.1 ± 17.4 (*n* = 32), average grip strength for the affected hand was 22.5 ± 14.8 kg (*n* = 39), average Arm and Leg MI score for the affected limb was 60.3 ± 28.6 (*n* = 24) and 64.9 ± 18.8 (*n* = 27), respectively, and average gait speed was 0.95 ± 0.31 m/s (*n* = 37).

### 3.3. Lesion Size and Motor Function

Total lesion volume was not significantly correlated (Spearman's rho) with motor function of the affected extremities as measured by BBT (*r* = −0.17, *p* = 0.29), grip strength (*r* = 0.10, *p* = 0.54), Arm MI (*r* = −0.15, *p* = 0.37), Leg MI (*r* = −0.06, *p* = 0.71), and gait speed (*r* = 0.15, *p* = 0.35).

### 3.4. FA/MD in A Priori ROIs and Motor Function

**Figure 1:**
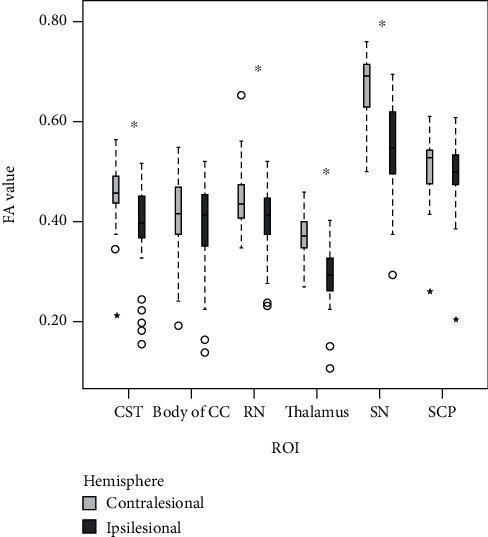
Differences in contralesional and ipsilesional structural integrity in a priori regions of interest (ROIs). Reduced fractional anisotropy (FA) values were observed in the ipsilesional hemisphere. The median FA value is represented by the solid black line; circles and stars represent outliers. The range lines indicate the limits of the first and third quartile of the interquartile range. CST: corticospinal tract; CC: corpus callosum; RN: red nucleus; SN: substantia nigra; SCP: superior cerebellar peduncle. ^∗^*p* < 0.01 for differences between sides.

FA values were significantly reduced in the ipsilesional hemisphere compared to the contralesional hemisphere in a priori ROIs (*p* < 0.01), except in the body of the corpus callosum (*p* = 0.20) and superior cerebellar peduncle (*p* = 0.06) (Figure 1). Correlations between upper/lower limb motor function and mean FA values are presented in Table 1. FA in the ipsilesional CST and RN was significantly correlated with motor function of the affected upper limb across all measures, with correlations ranging from 0.36-0.55. FA in the ipsilesional CST was significantly correlated with motor function of the affected lower limb (Leg MI_Aff_, *r* = 0.44), and FA in the ipsilesional RN was correlated with both Leg MI_Aff_ score (*r* = 0.49) and gait speed (*r* = 0.50). Additionally, FA in the ipsilesional substantia nigra was significantly correlated with Leg MI_Aff_ score (*r* = 0.37). No significant correlations were found between affected upper/lower limb motor function and mean FA values in contralesional ROIs.

A significant MD-behavior correlation was observed between the contralesional superior cerebellar peduncle and grip strength of the affected hand (*r* = −0.37, *p* = 0.008, *n* = 42) and Leg MI (*r* = −0.40, *p* = 0.005, *n* = 42). Otherwise, no significant MD correlations were found across behavioral measures.

For the exploratory analyses, the low functioning group showed similar trends to the overall analysis with stronger correlations demonstrated between ipsilesional CST FA and all upper limb measures (*r* = 0.71‐0.91, *p* < 0.001, *n* = 18‐20 depending on measure) and Leg MI_Aff_ (*r* = 0.72, *p* = 0.001, *n* = 15). FA in the ipsilesional RN, however, was only significantly correlated with BBT_Aff_ (*r* = 0.51, *p* = 0.01, *n* = 21). Contralesional CST FA was also significantly correlated with all upper limb measures and Leg MI_Aff_ (*r* = 0.61‐0.66, *p* ≤ 0.005, *n* = 16‐21 depending on measure), while FA in the contralesional body of the corpus callosum was significantly correlated with gait speed (*r* = 0.62, *p* = 0.009, *n* = 14). For the high functioning group, only FA in the ipsilesional body of the corpus callosum (*r* = −0.72, *n* = 18) and thalamus (*r* = −0.56, *n* = 18) were significantly correlated with grip strength (*p* ≤ 0.008); no other DTI metrics were significantly correlated with motor function in the high-functioning group.

### 3.5. Multiple Regression Analyses

Only the two ROIs with the strongest bivariate correlation with motor function were entered into the regression models due to our sample size. After controlling for significant covariates, ipsilesional CST FA was entered first into each model as the CST is the major neural pathway for skilled, discrete voluntary movements [18]. Ipsilesional RN FA was the next predictor entered into the models. The gait speed regression model did not include ipsilesional CST FA, however, as it was not significantly correlated with gait speed. A significant, positive correlation was found between ipsilesional CST and RN FA (Spearman′s *r* = 0.42, *p*  = 0.01, *n* = 42), yet since the correlation between these independent variables was fairly low, both variables were retained for analyses (Table 2). Ipsilesional CST FA significantly explained 37.3% of the variance in grip strength of the affected hand (*F*(1, 38) = 22.00, *p* < 0.001) and 31.5% of the variance in Arm MI_Aff_ score (*F*(1, 23) = 10.11, *p* = 0.004). Adding ipsilesional RN FA to the models, however, did not significantly explain an additional amount of variance in upper limb motor performance. No other regression analyses revealed significant models (at corrected *p* ≤ 0.01), but several models approached significance (at *p* ≤ 0.05). Multiple regression analyses were not conducted for the exploratory analyses due to the small sample sizes within each subgroup.

## 4. Discussion

This study examined relationships between structural integrity of different motor tracts/brain regions and affected upper and lower limb motor function in chronic stroke. Integrity of the ipsilesional CST, RN, thalamus, and substantia nigra was significantly lower compared to homologous regions in the contralesional hemisphere. Variability in the structural integrity of the ipsilesional CST not only correlated with affected upper limb motor function but also with lower limb strength; however, the CST appears to be less important for gait speed. Furthermore, structural integrity of the ipsilesional RN was associated with upper and lower limb motor performance, including gait speed, suggesting this region may contribute to motor outcome in chronic stroke. This suggestion is supported by findings that rubral branch integrity is associated with upper and lower limb motor impairment in chronic stroke [41, 42], as well as lesion studies in animals and neuroimaging studies in humans correlating the RN with motor function [43]. This study is novel in that it included both cortical and subcortical measures of structural integrity and examined brain-behavior relationships as it relates to both upper and lower limb motor function. Integrity of not only the CST but other brain structures involved in motor control may play a role in subsequent long-term motor outcome in chronic stroke [44–46].

Our findings provide further evidence that preservation of ipsilesional CST integrity is critical for chronic motor function poststroke [9, 12]. Less is known, however, about the contribution and microstructural changes of the contralesional CST after stroke. Schaechter et al. [47] found that FA in both the ipsilesional and contralesional CST was significantly and positively correlated with motor skill performance of patients' affected hand. Accumulating evidence indicates that white matter remodeling occurs in both ipsilesional and contralesional hemispheres, suggesting that structural remodeling of the contralesional motor system also contributes to motor recovery after stroke [3, 48]. Our results, however, did not find a significant correlation between contralesional CST FA and motor performance of the affected upper/lower limbs. This difference could be attributed to different sample characteristics (e.g., higher degree of motor impairment in previous studies compared to the current study) and/or to differences in measures of motor function.

FA in the ipsilesional RN was also associated with affected upper and lower limb motor function. Anisotropy within the RN has been postulated to be associated with its afferent and efferent fibers [16], such as the rubrospinal tract (RST). Animal studies have shown that the RST has connections with contralateral spinal motor neurons [49] and have suggested a compensatory role of the RST in motor recovery following CST injury in nonhuman primates [50]. Although the RST is more anatomically prominent in animals compared with humans [51], the RST may undergo neuroplastic changes poststroke that promotes increased functional contribution to motor recovery. Only a few studies, however, have examined neuroplastic changes in the RN following CST injury in humans after stroke. In persons 8-21 days poststroke, Yeo and Jang [13] found higher mean FA in the ipsilesional RN compared to the contralesional RN and healthy controls. Takenobu et al. [8] also found increased FA in the ipsilesional RN compared to the contralesional RN at 3 months poststroke, with a positive correlation with motor recovery. Rüber et al. [17] found higher FA values in both ipsilesional and contralesional RN in 18 chronic stroke patients compared to healthy controls, with significant positive correlations between red nuclei FA and level of motor function. These results suggest that remodeling and neuroplastic changes occur in the RN during the early stages of stroke and are still evident in the chronic stage and that these changes may indicate compensation for CST injury and contribute to motor outcome. Our results show that variability in upper and lower limb motor function in chronic stroke is associated with variability in ipsilesional RN FA, although we cannot directly infer a compensatory role of the RST in motor recovery. The human RN receives extensive input from the cerebral cortex and is involved in grasping, motor control, and coordination [43]. The RN may also play an important role in the neonatal brain, with regression in the adult brain linked to the development of upright bipedalism [52]. However, more clarity is needed regarding the function of the human RN and its role in motor recovery poststroke.

Of the other motor subnetwork a priori ROIs, only FA in the ipsilesional substantia nigra (SN) revealed a significant relationship with chronic motor performance (BBT_Aff_ and Leg MI_Aff_). The SN is part of the basal ganglia and is involved in motor control. It receives input from other basal nuclei, the cerebral cortex, and midbrain reticular formation; efferent information passes back to the cerebral cortex, basal ganglia, red nucleus, thalamus, and amygdala [15]. Our results suggest that ipsilesional SN integrity may also play a role in upper and lower limb motor outcome in chronic stroke.

In contrast to previous studies [8, 10, 36], we did not find significant correlations between the microstructural integrity of the corpus callosum and motor performance. This difference could be attributed to different sample characteristics (higher versus lower functioning), different motor assessments (e.g., prior studies commonly used the Fugl-Meyer assessment), and/or differences in how the corpus callosum was delineated or whether lesioned voxels were included in the analyses. We examined only the body of the corpus callosum, which contains the commissural fibers connecting bilateral motor cortices, using the JHU ROI atlas and only included ROIs with <5% lesion volume in our analyses.

Regression analyses revealed that ipsilesional CST FA was a predictor of grip strength and Arm MI_Aff_ score. These results complement other studies that have shown integrity of the ipsilesional CST [2, 36] or CST/PLIC FA asymmetry [9, 19] is related to upper limb motor performance poststroke. Adding ipsilesional RN FA to the models did not significantly explain more variance in motor performance. In terms of lower limb motor function, neither ipsilesional CST or RN FA were significant predictors. This finding may be a reflection of the complex neural circuitry involved with locomotion. The neural control of upper and lower limb movements is not analogous, as spinal interneurons play a role in the central pattern generation of gait [53] while fine hand movements are primarily under cerebral control. A few studies have suggested that the lateral CST does not play a central role in basic locomotor function in primates or humans [20], but rather is involved in gait adaptation [54]. Paretic leg movements and locomotor ability have also been present in some stroke survivors despite complete lateral CST injury [20, 21]. Other descending neural pathways such as the reticulospinal, rubrospinal, and vestibulospinal tracts could contribute to locomotor function and recovery after stroke. As stated previously, significant correlations were found between ipsilesional RN FA and lower limb motor performance in our study.

In our exploratory analyses examining brain-behavior relationships based on level of motor severity, the low functioning group exhibited similar trends (with stronger correlations) between ipsilesional CST FA and upper/lower limb motor function, providing further evidence that reduced structural damage to the ipsilesional CST is important for motor recovery in individuals with more severe motor impairment [9, 55, 56]. Ipsilesional RN FA was still correlated with BBT_Aff_ but was no longer correlated with grip strength, Motricity Index, or gait speed at the corrected *p* value of ≤0.01. Additionally, contralesional CST FA correlated with upper limb motor function across all measures. This finding is in line with prior work suggesting that the contralesional CST plays a compensatory role to support paretic upper limb movements in individuals with more severe motor deficits [47, 57, 58]. In contrast, only FA in the ipsilesional body of the corpus callosum and thalamus correlated with paretic hand function in the high functioning group. Recent work has found that motor function in individuals with mild upper limb impairments is correlated with integrity of the corpus callosum but not the CST [59, 60], suggesting that interhemispheric structural connections between motor cortices play a supportive role in chronic motor recovery in this population. Regarding lower limb brain-behavior relationships, bilateral CST FA correlated with affected lower limb strength and FA in the contralesional body of the corpus callosum correlated with gait speed, but only in the low functioning group. Research has demonstrated less lateralization of cortical activity with leg movements compared to hand movements [61], which is not surprising as lower limb movements are mainly bilateral.

Regarding mean diffusivity, significant findings between MD and motor function were minimal. Only a few studies have reported significant relationships between white matter integrity (as indexed by MD) and sensorimotor function in chronic stroke [62–64], while others have found no relationship [65, 66]. Furthermore, while MD values can change quite rapidly after stroke, MD has been shown to be pseudonormalized within 96 hours poststroke in animals models [67, 68].

This study is one of the first to incorporate a large sample size with left hemisphere necrotic damage when examining the relationship between motor function and microstructural integrity in chronic stroke and may explain some of the differences with previous studies. As there are hemispheric differences in motor structure and activation related to motor attention, action selection, and task complexity [69, 70], examining changes in structural integrity following right hemispheric stroke and relationships to motor function is also imperative. Increasing our understanding of the relationship between structural integrity of multiple motor pathways and motor function poststroke may provide greater insight into mechanisms that support brain plasticity and motor recovery. Such information could improve motor recovery prognosis and assist with targeting therapeutic interventions. For example, researchers and clinicians could design treatment approaches that utilize different motor pathways/brain regions depending on structural integrity metrics of specific motor pathways. Associations between upper limb motor impairment and microstructural integrity of the CST [9], transcallosal tracts [10], and extrapyramidal pathways (rubrospinal and reticulospinal) [42] in chronic stroke highlight the importance of examining multiple motor pathways/brain regions as potential biomarkers for motor outcome and response to treatment. Future studies examining training-induced neuroplastic changes are also warranted as treatment-related white matter changes (i.e., increase in FA pre-posttreatment) have been associated with both speech [71] and upper limb motor gains [72] in chronic stroke.

Our findings need to be interpreted in the context of our study design and the limitations of DTI and FA. The measured diffusion tensor is an average of several tissue compartments with different diffusion profiles within each voxel. Therefore, areas of partial tissue volume (mixture of white matter/gray matter/cerebrospinal fluid) or white matter partial volume (crossing or diverging fibers) will result in low anisotropy [73]. Furthermore, many factors influence FA values (e.g., axonal count/density and fiber organization) and DTI cannot discern which structural element(s) are contributing to observed changes in FA. Additionally, our ROIs were defined by the atlas we used; for example, we assessed only the distal portion of the CST. Other studies have used alternative analysis techniques that incorporate different ROI parameters, utilize a tract-based approach, or evaluate FA at different levels along motor tracts [9, 19, 36, 47]. While each method has its advantages and limitations, the different approaches are able to detect decreased FA values in persons poststroke and identify behavioral correlations [74], and the optimal approach for the investigation of brain-behavior relationships remains unclear. Further, using ROIs from an atlas developed in healthy adults is critically dependent on spatial normalization quality, such that larger lesions may cause more atrophy leading to a larger displacement of a region (though we note that we used state-of-the-art normalization methods). Lastly, our sample included a large number of subjects with mild motor impairment; results may not extrapolate to individuals with more severe impairment.

## 5. Conclusions

Our findings demonstrate that microstructural integrity of the ipsilesional CST is correlated with both affected upper and lower limb motor function across different ICF domains in chronic stroke. The CST appears to be less important, however, for gait speed than to the control of upper limb dexterity and strength. Additionally, microstructural integrity of the ipsilesional RN was fair to moderately associate with motor performance of the affected limbs, suggesting that this region may contribute to motor outcome poststroke. Ipsilesional RN FA, however, did not significantly explain an additional amount of variance in upper limb strength beyond that explained by ipsilesional CST FA. Further elucidation of the role that secondary motor pathways which plays in chronic motor function is needed, as well as relationships between structural and functional reorganization. Such insight could help with targeting rehabilitation techniques in an effort to optimize motor recovery following stroke.

## Figures and Tables

**Table 1:** Nonparametric correlations between motor function and mean FA values in a priori ROIs.

FA in *a priori* ROIs	Behavioral measures				
	BBT_Aff_ (*n* = 42)†	Grip_Aff_ (*n* = 42)†	Arm MI_Aff_ (*n* = 40)†	Leg MI_Aff_ (*n* = 42)†	Gait speed (*n* = 40)†
Ipsilesional					
CST	0.55^∗∗^ (*n* = 41)	0.38^∗^ (*n* = 41)	0.52^∗∗^ (*n* = 39)	0.44^∗^ (*n* = 41)	0.35 (*n* = 39)
Body of CC	0.05 (*n* = 34)	-0.25 (*n* = 34)	0.12 (*n* = 33)	-0.03 (*n* = 34)	-0.27 (*n* = 33)
Red nucleus	0.45^∗∗^	0.36^∗^	0.45^∗^	0.49^∗∗^	0.50^∗∗^
Thalamus	0.33 (*n* = 33)	0.11 (*n* = 33)	0.39 (*n* = 32)	0.14 (*n* = 34)	-0.12 (*n* = 32)
Substantia nigra	0.36^∗^ (*n* = 41)	0.19 (*n* = 41)	0.36 (*n* = 39)	0.37^∗^ (*n* = 41)	0.17 (*n* = 39)
SCP	0.10	0.17	0.19	0.26	0.20
Contralesional					
CST	0.13	0.23	0.15	0.11	0.11
Body of CC	0.11	-0.05	0.10	0.01	-0.04
Red nucleus	0.01	-0.08	0.06	0.08	0.22
Thalamus	0.02	0.07	0.02	-0.02	-0.19
Substantia nigra	-0.25	-0.24	-0.26	-0.25	-0.15
SCP	0.09	0.26	0.18	0.30	0.36

Values in the table are Spearman's coefficients (*r*). Abbreviations: ROIs: regions of interest; BBT_Aff_: Box and Block Test (affected limb); Grip_Aff_: grip strength (affected limb); Arm MI_Aff_: Arm Motricity Index score (affected limb); Leg MI_Aff_: Leg Motricity Index score (affected limb); CST: corticospinal tract; CC: corpus callosum; SCP: superior cerebellar peduncle. †Overall *n* value (*n* exceptions noted in parentheses, accounting for ROIs with ≥5% lesion volume). ^∗∗^*p* ≤ 0.001; ^∗^*p* ≤ 0.01.

**Table 2:** Multiple regression analyses of tract/region-specific FA and upper and lower limb motor performance.

Behavioral measure	Predictors	*R* ^2^	*F* statistic	*p* value for *F*	*β*_TSS_	*β*_CST_	*β*_RN_
BBT_Aff_ (*n* = 32)^a^							
Model 1	TSS, FA CST_ipsi_	0.264	5.21	0.012	-0.258	0.335	
Model 2	TSS, FA CST_ipsi_, FA RN_ipsi_	0.270	3.45	0.030	-0.230	0.344	0.076
Grip_Aff_ (*n* = 39)^a^							
Model 1	FA CST_ipsi_	0.373	22.03	≤0.001†		0.611^∗∗^	
Model 2	FA CST_ipsi_, FA RN_ipsi_	0.374	10.74	≤0.001†		0.597^∗∗^	0.027
Arm MI_Aff_ (*n* = 24)^b^							
Model 1	FA CST_ipsi_	0.315	10.13	0.004†		-0.561^∗∗^	
Model 2	FA CST_ipsi_, FA RN_ipsi_	0.337	5.34	0.013		-0.472^∗^	-0.173
Leg MI_Aff_ (*n* = 27)							
Model 1	FA CST_ipsi_	0.186	5.72	0.025		0.432^∗^	
Model 2	FA CST_ipsi_, FA RN_ipsi_	0.241	3.82	0.036		0.282	0.278
Gait speed (*n* = 39)^c^							
Model 1	TSS, FA RN_ipsi_	0.237	5.82	0.021	-0.239	—	0.365∗

Abbreviations: BBT_Aff_: Box and Block Test (affected limb); Grip_Aff_: grip strength (affected limb); Arm MI_Aff_: Arm Motricity Index score (affected limb); Leg MI_Aff_: Leg Motricity Index score (affected limb); TSS: time since stroke; FA: fractional anisotropy; CST: corticospinal tract; RN: red nucleus. †Significant at corrected *p* ≤ 0.01. ^∗∗^*p* ≤ 0.01; ^∗^*p* ≤ 0.05; ª2 outliers removed; ^b^Arm MI_Aff_ behavioral scores reflect and square root transformed; ^c^1 outlier removed.

## Data Availability

Data are available from the corresponding author upon request.
